# Interpreting ciliopathy-associated missense variants of uncertain significance (VUS) in *Caenorhabditis elegans*

**DOI:** 10.1093/hmg/ddab344

**Published:** 2021-11-20

**Authors:** Karen I Lange, Sunayna Best, Sofia Tsiropoulou, Ian Berry, Colin A Johnson, Oliver E Blacque

**Affiliations:** School of Biomolecular and Biomedical Science, University College Dublin, Belfield, Dublin 4, Ireland; Division of Molecular Medicine, Leeds Institute of Medical Research, University of Leeds, Leeds, West Yorkshire, UK; School of Biomolecular and Biomedical Science, University College Dublin, Belfield, Dublin 4, Ireland; Bristol Genetics Laboratory, Pathology Sciences, Southmead Hospital, Bristol BS10 5NB, UK; Division of Molecular Medicine, Leeds Institute of Medical Research, University of Leeds, Leeds, West Yorkshire, UK; School of Biomolecular and Biomedical Science, University College Dublin, Belfield, Dublin 4, Ireland

## Abstract

Better methods are required to interpret the pathogenicity of disease-associated variants of uncertain significance (VUS), which cannot be actioned clinically. In this study, we explore the use of an animal model (*Caenorhabditis elegans*) for *in vivo* interpretation of missense VUS alleles of *TMEM67*, a cilia gene associated with ciliopathies. CRISPR/Cas9 gene editing was used to generate homozygous knock-in *C. elegans* worm strains carrying *TMEM67* patient variants engineered into the orthologous gene (*mks-3*). Quantitative phenotypic assays of sensory cilia structure and function (neuronal dye filling, roaming and chemotaxis assays) measured how the variants impacted *mks-3* gene function. Effects of the variants on *mks-3* function were further investigated by looking at MKS-3::GFP localization and cilia ultrastructure. The quantitative assays in *C. elegans* accurately distinguished between known benign (Asp359Glu, Thr360Ala) and known pathogenic (Glu361Ter, Gln376Pro) variants. Analysis of eight missense VUS generated evidence that three are benign (Cys173Arg, Thr176Ile and Gly979Arg) and five are pathogenic (Cys170Tyr, His782Arg, Gly786Glu, His790Arg and Ser961Tyr). Results from worms were validated by a genetic complementation assay in a human *TMEM67* knock-out hTERT-RPE1 cell line that tests a *TMEM67* signalling function. We conclude that efficient genome editing and quantitative functional assays in *C. elegans* make it a tractable *in vivo* animal model for rapid, cost-effective interpretation of ciliopathy-associated missense VUS alleles.

## Introduction

Exome and genome sequencing have revolutionized our ability to identify the genetic causes of disease, interrogate disease mechanisms and pinpoint gene targets for therapy. Missense variants (single codon altered to encode a different amino acid) are the most numerous class of protein-altering variants (1) but only a subset are associated with disease (2). Based on disease features and patterns of inheritance, identified variants are classified as benign, likely benign, uncertain significance, likely pathogenic or pathogenic (as defined by the Association for Clinical Genomic Science; ACGS) (3). For novel or previously uncharacterized variants, the only evidence available to assess their pathogenicity is population allele frequency and analysis by *in silico* tools (e.g. SIFT/PolyPhen/CADD), which are not sufficient to meet the threshold for a ‘likely pathogenic’ classification according to best practices established by ACGS (3). A VUS (variant of uncertain significance, VUS) classification is made when there is insufficient evidence to conclude on pathogenicity (3,4). Currently, most missense variants are classified as VUS (118 864/206 594 = 57.5%, accessed from ClinVar (5) November 2021). Since VUS designations cannot be acted upon clinically, a VUS classification can delay or prohibit accurate disease management and/or genetic counselling, and prevent patients from accessing gene-specific therapies and clinical trials (6,7). Given the pressing clinical need to reclassify VUS as benign or pathogenic, it is clear that new effective experimental strategies for VUS interpretation are required (8). With the emergence of advanced genetics tools such as CRISPR-Cas9 gene editing, non-rodent model organisms such as zebrafish, *Drosophila* and *C. elegans* are emerging as robust *in vivo* experimental platforms for functional interpretation of variant pathogenicity (9–12).

Ciliopathies are a heterogenous group of at least 25 inherited disorders with clinically overlapping phenotypes, caused by pathogenic variants in >200 genes (13,14). Ciliopathies affect many organ systems, causing a broad range of clinical phenotypes of varied severity and penetrance that include cystic kidneys, retinal dystrophy, bone abnormalities, organ laterality defects, respiratory tract defects, infertility, obesity, neurodevelopmental defects and cognitive impairment (15). Due to the extreme heterogeneity of ciliopathy phenotypes, it can be difficult to accurately diagnose ciliopathies. For example, in the UK 100 000 genomes project, >20% of patients recruited in the ciliopathy cohort were subsequently diagnosed with non-ciliopathy disorders (14). Several gene therapies targeting ciliopathy genes are currently in development (16–19), highlighting the need to increase accurate genetic diagnoses for these disorders. Ciliopathies are caused by defects in cilia which are 2–20 micron-long microtubule-based organelles that extend from the surfaces of most cell types. Motile cilia propel cells through a fluid or push fluid across a tissue surface. Primary cilia act as cellular ‘antennae’ (20), transducing a wide variety of extrinsic chemical and physical (e.g. light, odorants) signals into the cell (21). Primary cilia are also especially important for coordinating cell–cell communication signalling pathways (e.g. Shh, Wnt, PDGF-a) that are essential for development and homeostasis (22).

The nematode *Caenorhabditis elegans (C. elegans)* is a leading model for investigating cilia biology, with many ciliopathy genes and associated pathways conserved in worms (23,24). In *C. elegans*, primary cilia are only found on 60 sensory neurons, extending from the distal tips of dendrite processes. Most nematode cilia are found in the animal’s head and are environmentally exposed via pores in the nematode cuticle (25). Recently, we used CRISPR-Cas9 knock-in technology and quantitative readouts of gene function to show that pathogenic missense mutations in the Joubert syndrome gene B9D2 are also pathogenic in the context of the *C. elegans* orthologue (26). Having established this proof-of-principle for modelling ciliopathy variants in worms, we have now examined the use of *C. elegans* for interpreting ciliopathy missense VUS. In this study, we have focussed on *TMEM67* (also called *MKS3*), which is associated with several ciliopathies including Meckel Syndrome (27–30) (OMIM #607361), COACH Syndrome (31) (OMIM #216360) and Joubert Syndrome (7,32–35) (OMIM #610688). Most missense variants reported in *TMEM67* have uncertain clinical significance (74/142 = 52.1%, accessed from ClinVar (5) November 2021), and their abundance makes *TMEM67* an excellent candidate to explore VUS interpretation in *C. elegans*.

TMEM67/MKS3 is a transmembrane protein that functions at the ciliary transition zone (TZ), which corresponds to the proximal-most 0.2–1.0 μm of the ciliary axoneme (27,36). Defined by unique structural features such as Y-linkers that connect the ciliary microtubules with the membrane, the TZ acts as a diffusion barrier, or ‘gate’, to facilitate the cilium as a compartmentalized organelle (37). Indeed, the TZ is a ciliopathy hotspot, with at least two dozen ciliopathy proteins found there (38). Work from multiple model systems, including major input from the nematode system, has identified several ciliopathy-associated genetic and molecular assemblies within the TZ such as the MKS and NPHP modules (39–41). In *C. elegans*, the TMEM67 orthologue MKS-3 forms part of the MKS module, along with at least 10 other ciliopathy proteins, whereas the NPHP module consists of just two proteins (NPHP-1, NPHP-4) (38). The relationship between the NPHP and MKS modules varies between organisms. *Drosophila* has a simplified TZ organization that lacks the NPHP module (42,43). Whereas *C. elegans* MKS and NPHP module genes function redundantly to regulate cilia and TZ formation (44), this is typically not the case in vertebrates and mammals where loss of individual MKS or NPHP module components, such as TMEM67, results in mild to severe ciliogenesis defects and lethality in some cases (45–52). Although the MKS and NPHP module genes are not strictly redundant in vertebrates, they do genetically interact (48,53)*.*

Here, we used CRISPR-Cas9 technology to engineer eight *TMEM67* missense VUS at the orthologous position in the *C. elegans mks-3* orthologue. Using quantifiable assays of cilium structure and sensory function, as well as protein localization at the TZ, we determined that three of the variants are benign and five are damaging. We then validated the worm findings using a genetic complementation-based approach in *TMEM67* null human cells. Our study indicates that *C. elegans* is a tractable model system that can provide evidence of pathogenicity for ciliopathy-associated variants.

## Results

### Selection of *TMEM67* variants for analysis

**Figure 1:**
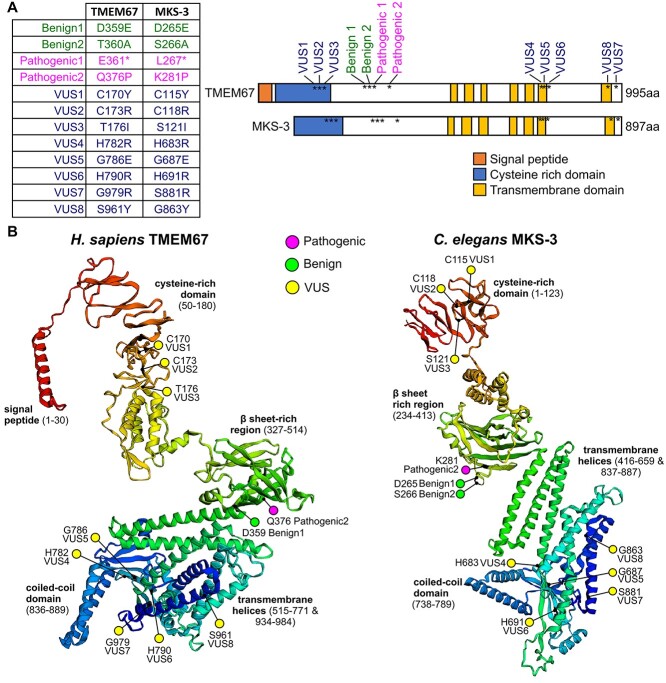
*TMEM67* variants analysed in this study. (**A**) Twelve variants in *TMEM67/mks-3* were generated by CRISPR-Cas9 gene editing and characterized in *C. elegans*. The schematic shows the relative positions of the variants along the length of the proteins. The domains are conserved between humans and worms, but the worm protein lacks the N-terminal signal peptide. (**B**) RaptorX protein structure and domain organization predictions for the full-length TMEM67 and MKS-3 proteins. RaptorX is a deep learning algorithm that predicts secondary and tertiary structures of proteins without close homologues or known structures in the protein data bank. Ribbon diagrams of proteins are rainbow-coloured (red at N-terminus to dark blue at C-terminus) with variants indicated (magenta—known pathogenic; yellow—VUS; green—known benign) within the predicted protein domains. Note that transmembrane helices 1–5 (green to cyan) are followed by a single coiled-coil domain (light blue) and then two C-terminal transmembrane helices 6–7 (dark blue).

TMEM67 variants were selected using two criteria: (i) conservation of the mutated amino acid in the worm orthologue (MKS-3) (Supplementary Material, Fig. S1), and (ii) presence of an adjacent Cas9 PAM site in the *C. elegans* genome to facilitate CRISPR gene editing. Using these criteria, we modelled eight missense *TMEM67* VUS in *C. elegans mks-3* (Fig. 1A**,**  Supplementary Material, Table S1). We also included two known benign and two pathogenic variants as controls. Benign1(Asp359Glu) and VUS4(His782Arg) were identified on the Ensembl variation database (54). Benign2(Thr360Ala), Pathogenic1(Glu361Ter), Pathogenic2(Gln376Pro), VUS2(Cys173Arg) and VUS5(Gly786Glu) were identified on ClinVar (5). VUS1(Cys170Tyr), VUS3(Thr176Ile), VUS6(His790Arg), VUS7(Gly979Arg) and VUS8(Ser961Tyr) were identified from clinical exome sequencing of Meckel syndrome foetuses. All variants analysed in this study are recessive alleles. For simplicity, we refer to the variants using a shorthand notation (e.g. Benign1, Pathogenic1, VUS1). A *de novo* protein structure prediction program, Raptor X, revealed that the human and worm TMEM67 proteins show remarkable similarity in their overall predicted domain organization and secondary structure (Fig. 1B). The targeted pathogenic, benign and VUS residues are present in comparable regions of secondary structure (Fig. 1B). For example, the known benign variant residues are in exposed loops and the known pathogenic residues are buried in $\beta$-sheets (Fig. 1B, Supplementary Material, Fig. S2).

### Quantitative phenotypic analysis of *mks-3* VUS alleles in *C. elegans*

We employed a CRISPR/Cas9 genome editing strategy to engineer homozygous *mks-3* mutant worms. In *C. elegans,* many TZ proteins belong to two genetically redundant entities termed the ‘MKS’ and ‘NPHP’ modules that regulate cilia formation and function (44). Relatively minor cilia-dependent phenotypes are observed in *mks-3* and *nphp-4* single mutants (such as a subtle chemotaxis defect), whereas severe cilia defects are observed in *mks-3; nphp-4* double mutants (36,55,56). Since *mks-3* functions redundantly with NPHP module genes, we generated the *mks-3* knock-in variants in an *nphp-4(tm925)* mutant background to facilitate phenotypic analysis. *nphp-4(tm925)* is a 1109 bp deletion, subsequently referred to as *nphp-4(∆).* In phenotypic assays, double mutant *mks-3(variant)*; *nphp-4(∆)* phenotypes were compared to *mks-3(+); nphp-4(∆)* (wild-type, positive control) and *mks-3(∆)*; *nphp-4(∆)* (949 bp deletion of *mks-3*, negative control). We hypothesized that pathogenic *mks-3* patient alleles would be phenotypically similar to the *mks-3(∆)* allele.

**Figure 2:**
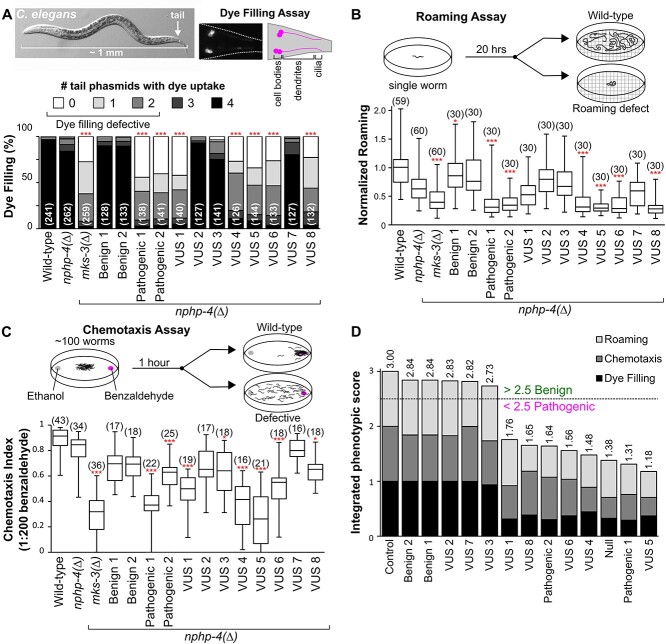
Quantitative phenotyping of cilia-dependent phenotypes in *C. elegans.* All assays were performed blind with at least three independent biological replicates. (**A**) Lipophilic dye (DiI or DiO) filling assay of the four phasmid (tail) neurons. The number of cell bodies which uptake dye was counted (values range from 0 to 4). The bar graph indicates the proportion of the population with dye uptake in 0 (white) to 4 (black) phasmid neurons. The number of worms is shown in brackets. Statistical significance according to Kruskal–Wallis followed by Schaich–Hammerle *post hoc* test. (**B**) Assessment of worm roaming behaviour normalized to wild-type. A single young adult hermaphrodite was placed on a food-rich plate for 20 h and the roaming activity was quantified. The number of worms is shown in brackets. Box plots indicate the maximum and minimum values (bars), median, lower quartile and upper quartile. Statistical significance according to Kruskal–Wallis followed by Dunn’s *post hoc* test. (**C**) Quantification of worm chemotaxis towards benzaldehyde after 60 min. Assay is performed on a population of 50–300 worms. The number of assays is shown in brackets. Statistical significance according to ANOVA followed by Tukey’s *post hoc* test. Box plots indicate the maximum and minimum values (bars), median, lower quartile and upper quartile. ^*^ and ^*^^*^^*^ refer to *P*-values of <0.05 and <0.001, respectively. (**D**) Integration of the phenotypic results from the three quantitative assays (panels A–C) into one value. Averages from each assay were normalized to the *nphp-4(∆)* control (with a maximum score of 1.0 for each assay) and summed. Values were ranked from highest (benign) to lowest (pathogenic).

To assess cilia structure and function we performed three quantitative assays: neuronal dye filling, roaming/foraging and chemotaxis. The dye filling assay indirectly assesses the structural integrity of cilia that are exposed to the environment via their location in head and tail sensory pores (57,58). Specifically, we assessed lipophilic dye (DiI/DiO) uptake in the four ciliated phasmid sensory neurons in the tail. Wild-type and *mks-3(+)*; *nphp-4(∆)* positive controls display robust dye filling, whereas the *mks-3(∆); nphp-4(∆)* negative control is dye filling defective (Fig. 2A). Benign1 and Benign2-containing strains show robust dye uptake whereas strains with Pathogenic1 and Pathogenic2 are defective (Fig. 2A). For strains with the VUS alleles, five cause a severe dye filling defect (VUS1/4/5/6/8), whereas three (VUS2/3/7) do not (Fig. 2A).

*C. elegans* foraging behaviour is dependent on sensory cilia (57,59). A single young adult worm is placed on a lawn of bacteria for 20 h and the extent of its roaming across the plate is quantified (Fig. 2B). *mks-3(+); nphp-4(∆)* positive control worms show a slight decrease in roaming compared to wild-type worms, whereas *mks-3(∆)*; *nphp-4(∆)* negative controls exhibit a severe roaming defect (Fig. 2B). As expected, Benign1 and Benign2-containing strains exhibit normal roaming behaviour, whereas Pathogenic1 and Pathogenic2-containing worm strains are roaming defective (Fig. 2B). For strains with the VUS alleles, those with VUS4/5/6/8 exhibit a roaming defect, whereas those with VUS1/2/3/7 are roaming normal (Fig. 2B).

*C. elegans* chemotaxis towards benzaldehyde is also dependent on sensory cilia (57,60). A population of 50–300 worms is placed in the centre of a plate, equidistant from spots of control (ethanol) and benzaldehyde (1:200 in ethanol). *mks-3(+); nphp-4(∆)* positive control worms show a slight reduction in chemotaxis compared to wild-type, whereas *mks-3(∆); nphp-4(∆)* negative controls exhibit a chemotaxis defective phenotype (Fig. 2C). As expected, Benign1 and Benign2-containing strains are chemotaxis normal whereas Pathogenic1 and Pathogenic2-containing strains are defective (Fig. 2C). For the VUS-containing strains, those with VUS1/4/5/6 show a severe chemotaxis defect, those with VUS3/8 exhibit an intermediate phenotype, and those with VUS2/7 are chemotaxis normal (Fig. 2C).

To derive a predictive ‘interpretation’ score for the VUS alleles, we integrated the results from the three phenotypic assays into a single value (equal weighting; averages normalized to the *mks-3(+); nphp-4(∆)* positive control; maximum score of 1.0 per assay) (Fig. 2D). A score of <2.5 is considered pathogenic. The Benign1 and Benign2 variants score similarly to the *mks-3(+); nphp-4(∆)* positive control, whereas Pathogenic1 and Pathogenic2 variants score similarly to the *mks-3(∆); nphp-4(∆)* negative control (Fig. 2D). Strains with VUS2, VUS3, or VUS7 received high scores comparable to the benign variants, whereas those with VUS1, VUS4, VUS5, VUS6 or VUS8 received scores comparable to the pathogenic variants. Therefore, in *C. elegans*, we conclude that VUS2/3/7 are benign variants and VUS1/4/5/6/8 are pathogenic variants.

### Effect of VUSs on TZ localization of MKS-3 and cilia ultrastructure

**Figure 3:**
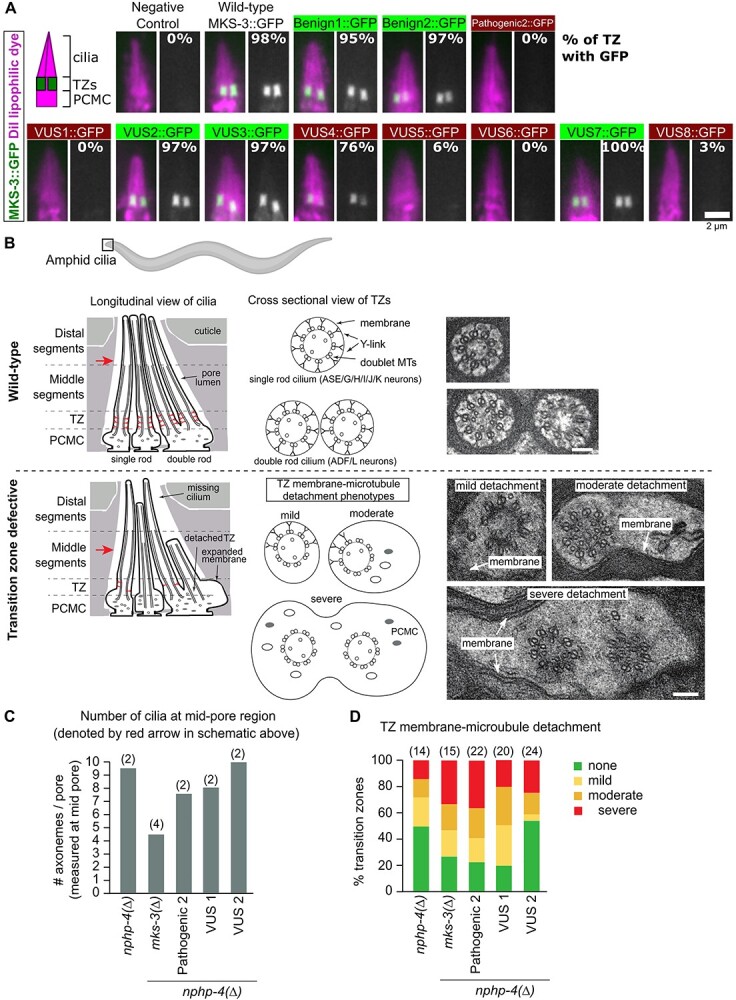
Testing *mks-3* variant predictions via analysis of GFP reporter localization and cilia/transition zone ultrastructure. (**A**) Transgenic worm strains containing MKS-3::GFP extrachromosomal arrays were generated in the *mks-3(Δ)* genetic background. DiI (magenta) lipophilic dye stains the cilia and periciliary membrane compartment (PCMC). Grayscale image shows the green channel alone. GFP levels from 34 to 40 transition zone (TZ) pairs were quantified for each variant. The percentage of cilia with MKS-3::GFP localized to the TZ is indicated. Scale bar is 2 μm. (B–D) Ultrastructure of amphid channel ciliary axonemes and the TZ compartment. TEM images in (**B**) show examples of wild-type and disrupted TZs, in cross section, for neurons with single and double rod cilia (scale bar is 100 nm). Disrupted TZs show loss of Y-links, resulting in varying degrees of microtubule detachment from the membrane, along with frequent expansion of the membrane. Mild—slight detachment of TZ microtubules from the membrane, which is expanded to a small degree; moderate—many TZ microtubules are detached from the membrane, which is extensively expanded; severe—most or all of the TZ microtubules are detached from the membrane, which is highly expanded, indicating ectopic docking of the TZ within the PCMC cytoplasm. Schematics show the amphid channel pore and cilia in longitudinal orientation (only 4 of the 10 ciliary axonemes are shown for simplicity), and TZs in radial orientation (cross-section), and indicate the phenotypes shown in the TEM images. The histogram in (**C**) shows the mean number of cilia observed in electron micrographs from cross sections of the mid-pore region (red arrows in B) taken from the indicated genotypes (number of pores analysed shown in brackets). The chart in (**D**) shows the quantification of the TZ membrane-microtubule detachment phenotypes for the indicated genotypes (number of TZs analysed shown in brackets).

To provide further insight into the damaging or benign nature of the *TMEM67* VUS alleles, we first examined the effect of the variants on the subcellular localization of MKS-3. In *C. elegans* sensory neurons, transmembrane MKS-3 localizes to the ciliary TZ, which corresponds to the most proximal ~1 μm of the ciliary axoneme adjacent to the basal body (36). Fusion PCR was used to generate linear *mks-3::gfp* fragments (Supplementary Material, Fig. S3A) containing each of the engineered variants. Constructs were then expressed as extrachromosomal arrays in *C. elegans*, and a fluorescent lipophilic red dye (DiI) employed to co-stain the ciliary membrane. Pathogenic1 was excluded from this analysis because it is a nonsense allele with a premature stop codon. As expected, MKS-3(+)::GFP, Benign1::GFP, and Benign2::GFP exhibit very specific TZ localization in the sensory neurons (Fig. 3A, Supplementary Material, Fig. S3B). In contrast, Pathogenic2::GFP showed no detectable fluorescence at the TZ, consistent with the finding that the human Pathogenic2 variant (Q376P) disrupts TMEM67 plasma membrane localization in cell culture (52). Our conclusion that VUS2, VUS3 and VUS7 are benign (Fig. 2D) predicts that the proteins should localize normally. Consistent with this hypothesis,VUS2::GFP, VUS3::GFP and VUS7::GFP display robust TZ localizations in transgenic worms, although Benign2 and VUS7 do show some modest reduction in signal levels (Fig. 3A, Supplementary Material, Fig. S3B). In contrast, VUS1::GFP, VUS5::GFP, VUS6::GFP and VUS8::GFP show no detectable TZ-localization, consistent with these variants being pathogenic. Mislocalized GFP signal elsewhere in the neurons is not observed for these proposed pathogenic VUS suggesting that these variant proteins may be misfolded, unstable and/or degraded. Interestingly, despite a predicted pathogenic classification (Fig. 2D), VUS4::GFP was TZ-localized in most transgenic worms, although signal levels were reduced by ~50% (Fig. 3A, Supplementary Material, Fig. S3B). This observation highlights that TZ-localization alone is not sufficient to interpret pathogenicity.

Using serial section transmission electron microscopy (TEM), we also assessed the effects of the TMEM67 VUS variants on the ultrastructure of amphid neuron cilia and TZs in the nose of the worms. Specifically, we analysed cross-sections of one predicted pathogenic VUS (*mks-3(VUS1)*), one predicted benign VUS (*mks-3(VUS2)*), along with positive (*mks-3(+)*) and negative (*mks-3(∆)*, *mks-3(Pathogenic2)*) controls. All strains were also homozygous for the *nphp-4(∆)* allele. Wild-type amphidal pores contain 10 rod-shaped ciliary axonemes emanating from the dendritic tips of eight sensory neurons (two neurons possess a pair of rods); each axoneme consists of middle (doublet microtubules) and distal (singlet microtubules) segments, and a proximal TZ compartment that emerges from a swelling at the distal dendrite tip called the periciliary membrane compartment (PCMC) (Fig. 3B). Analysis of cross sections taken from the mid region of the pore shows that *nphp-4(∆)* worms containing *mks-3(+)* or *mks-3(VUS2)* display an almost full complement of 10 cilia. In contrast, at least two axonemes are missing in the corresponding cross sections of *nphp-4(∆)* worms with *mks-3(∆), mks-3(Pathogenic2)* or *mks-3(VUS1)*, indicating that some axonemes are either truncated or missing entirely in these strains (Fig. 3C). In cross-sections of the TZ and PCMC regions, ~50% of the TZs of *nphp-4(∆)* worms with *mks-3(+)* or *mks-3(VUS2)* show a wild-type phenotype, where the TZ membrane and microtubules are in close apposition, connected by electron dense Y-linkers (Fig. 3B). In contrast, *nphp-4(∆)* worms with *mks-3(∆)*, *mks-3(Pathogenic2)* or *mks-3(VUS1)* show a more disrupted phenotype, with severe loss of Y-linkers and expansion of the surrounding ciliary/periciliary membrane (Fig. 3D). Together, the ultrastructure data for axoneme number and TZ integrity shows that VUS1 phenocopies the defects observed in the negative controls, whereas VUS2 phenocopies the positive control, thereby confirming the pathogenic and benign nature of VUS1 and VUS2, respectively.

**Figure 4:**
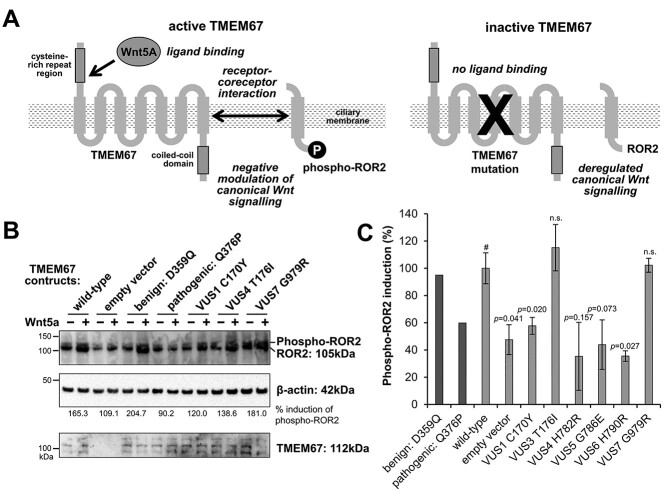
Validation of *C. elegans* predictions of TMEM67 VUS pathogenicity in cell culture. (**A**) Schematic summarizes the genetic complementation assay in hTERT-RPE1 *TMEM67* knock-out cells. Left: in the presence of TMEM67, phosphorylation of the co-receptor ROR2 is stimulated by exogenous treatment with the non-canonical ligand Wnt5a in comparison to control treatment. Right: if TMEM67 is lost or disrupted, ROR2 phosphorylation is not stimulated by this treatment. (**B**) Western blots of ROR2, with upper phosphorylated isoform indicated (top panel), following transfection and expression (bottom panel) of TMEM67 constructs (wild-type, empty vector negative control, known benign variant control, known pathogenic variant control, and a selection of VUS alleles). Transfected *TMEM67^-/-^* knock-out cells were treated with control conditioned medium (−) or Wnt5a-conditioned medium (+). Loading control for normalization is $\beta$-actin. (**C**) Densitometry scans of the phosphorylated ROR2 isoform (for *n* = 3 biological replicates) were quantitated in the bar graph for percentage induction of Wnt5a-stimulated response compared to control response, normalized against responses to the wild-type TMEM67 construct. Statistical significance was determined in pairwise t-tests with wild-type (#) for a minimum of *n* = 3 biological replicates. *P*-values are listed, n.s., not significant. Error bars indicate standard error of the mean. Statistical significance is not included for Benign(D359Q) and Pathogenic(Q376P) because these values were derived from a single biological replicate.

### *In vitro* genetic complementation assay of TMEM67 VUS function in human cell culture

To further validate our findings from *C. elegans*, we utilized an *in vitro* human cell culture-based assay of *TMEM67* function. Previously, we demonstrated that TMEM67 is required for phosphorylation of the ROR2 co-receptor and subsequent activation of non-canonical Wnt signalling (61) (Fig. 4A). Here, we developed a genetic complementation approach using a validated hTERT-RPE1 crispant cell-line that has compound heterozygous (biallelic) null mutations in *TMEM67* (Supplementary Material, Fig. S4). In the absence of TMEM67, phosphorylation of ROR2 was not stimulated by exogenous treatment with the non-canonical ligand Wnt5a. Transient transfection with full-length wild-type TMEM67 fully rescued ROR2 phosphorylation following Wnt5a treatment (Fig. 4B). These responses allowed us to determine the relative effects of VUS on TMEM67 biological function. In this assay, transfection of Benign1 allowed 204.7% induction of phospho-ROR2 levels by Wnt5a relative to control (Fig. 4B). In contrast, Pathogenic2 did not rescue biological function (90.2% induction) (Fig. 4B). Comparison of all VUS, normalized to wild-type TMEM67 responses across three independent biological replicates, enabled us to interpret VUS1, VUS4, VUS5 and VUS6 as pathogenic, and VUS3 and VUS7 as benign (Fig. 4C). VUS2 and VUS8 were not tested in this assay. Thus, our predictions for VUS pathogenicity in *C. elegans* are corroborated by similar findings in mammalian cells.

## Discussion

Ciliopathies are multisystem disorders that affect many organs including kidneys, liver and retina. Although the organ systems affected by cilia dysfunction are not present in *C. elegans*, the basic biology of primary cilia is shared across species. Furthermore, despite undoubted context-specific distinctions, the cilia proteins themselves are functionally conserved (23,24), therefore allowing us to model and characterize patient missense variants in worms.

In this study, we exploited efficient genome editing and quantitative phenotypic analysis of cilium structure and function in *C. elegans* to determine the pathogenicity of *TMEM67* variants. This approach accurately classified known pathogenic and known benign variants. We also generated a pathogenicity prediction for all eight missense VUS alleles analysed. Three VUS were phenotypically benign (VUS2(Cys173Arg), VUS3(Thr176Ile), VUS7(Gly979Arg)) and five were phenotypically pathogenic (VUS1(Cys170Tyr), VUS4(His782Arg), VUS5(Gly786Glu), VUS6(His790Arg), VUS8(Ser961Tyr)). We validated these predictions using TEM and localization assay data, showing that pathogenic missense mutations abrogate TZ ultrastructure and prevent MKS-3 from localizing at the TZ. The one exception was VUS4, which although severely pathogenic for cilium structure and function, can localize at the TZ, albeit at a reduced level. Thus, the VUS4 patho-mechanism is likely due to loss of function at the TZ (such as disrupting a protein–protein interaction), rather than disruption of upstream MKS-3 trafficking to the compartment. Finally, we validated our nematode-based predictions in a human cell culture assay of TMEM67 function. When taken together, our data show that *C. elegans* can interpret the pathogenicity of VUS and provide evidence towards their reclassification as benign or pathogenic.

Several *in silico* algorithms have been developed to predict the pathogenicity of missense variants. However, their accuracy is inconsistent (62–64). Indeed, we tested five *in silico* tools (MISTIC (65), SIFT (66), Poly-Phen (67), CADD (68) and REVEL (69)) and found that they return deleterious/damaging predictions for all eight of the VUS examined in this project (Supplementary Material, Fig. S5). The only exceptions are VUS1/3/8, where one or two of the tools returned non-deleterious or intermediate scores (Supplementary Material, Fig. S5). Therefore, the algorithm predictions do not align with our observations in *C. elegans* and cell culture experiments. We conclude that *in vivo* modelling of missense variants in *C. elegans* more accurately predicts results in human cells than currently available prediction algorithms.

An additional advantage of using nematodes to interpret genetic variation is the availability of quantitative assays that are suitable for high-throughput analysis. For example, live animal fluorescence-activated cell sorting (FACS) can be used for assessing cilium structure (via dye filling) and automated worm tracking can be applied to measure cilium function (roaming, chemotaxis) (70–72). Furthermore, machine learning can streamline the analysis of complex datasets to predict VUS pathogenicity (73). Another advantage of the nematode approach is that engineered patient alleles can be used for high-throughput small molecule suppressor screens to identify potential therapeutics. Indeed, *C. elegans* is emerging as an excellent model for whole animal large-scale drug screens and such strategies have already been used to investigate a variety of metabolic and neuromuscular disorders (74–78)*.* Despite these advantages, one limitation to modelling patient variants in worms is that a substantial number of residues mutated in disease are not conserved in the *C. elegans* orthologue*.* However, a potential solution to this problem is to create ‘humanized’ worms, where the entirety or specific domains of the nematode orthologue are replaced with the human sequence (79,80). If the human protein retains functionality in the worm context, humanized strains can be used to model all missense human variants in the corresponding gene.

The utility of the nematode approach to interpreting human gene variants goes well beyond TMEM67 and the ciliopathy gene class. Indeed, a humanized nematode model was very recently employed to interpret the pathogenicity of 29 missense VUS in an epilepsy gene (73). Thus, for genes functioning in conserved molecular pathways, worms offer a powerful system to generate *in vivo* evidence towards reclassifying VUS as pathogenic or benign. With ever increasing throughput in generating the knock-in alleles, *C. elegans* can therefore make a significant contribution to interpreting the huge numbers of VUS deposited in ClinVar, and bring us closer to the ambition that the clinical relevance of all encountered genomic variants will be more readily predictable (81).

In summary, this study highlights that *C. elegans* is a practical model for variant interpretation of ciliary genes. Analysis of ciliopathy-associated VUS in *C. elegans* is accurate, quick, affordable and easily interpretable. Although this study focussed on TMEM67, we anticipate that VUS alleles of any conserved cilia genes can be modelled and characterized in *C. elegans* using the approach described here.

## Materials and Methods

### Modelling of protein secondary structure

Human TMEM67 (NP_714915.3) and *C. elegans* MKS-3 (NP_495591.2) protein sequences were analysed by the RaptorX protein structure prediction server, using default settings for the deep dilated convolutional residual neural networks method (82). Absolute model quality was assessed by ranking Global Distance Test (GDT) scores defined as 1×N(1)+0.75×N(2)+0.5×N(4)+0.25×N(8), where N(*x*) is the number of residues with estimated modelling error (in Å) smaller than *x*, divided by protein length and multiplied by 100. GDT scores >50 indicate a good quality model. However, the highest ranking models for TMEM67 (GDT = 28.968) and MKS-3 (GDT = 19.269) suggest that portions of these models are lower quality. Models in the .pdb format were visualized and annotated in EzMol (http://www.sbg.bio.ic.ac.uk/ezmol/).

### *C. elegans* maintenance

All *C. elegans* strains in this study were maintained at 20°C or 15°C on nematode growth medium (NGM) seeded with OP50 *E. coli* using standard techniques (83). Young adult hermaphrodites were synchronized by selecting L4 larvae and incubating at 20°C for 16–20 h or by alkaline hypochlorite treatment of gravid hermaphrodites at 20°C ~65–70 h before the assay. All worm strains are listed in Supplementary Material, Table S2.

### CRISPR/Cas9 to engineer *mks-3* mutants in *C. elegans*

CRISPR protocols were performed as previously described (26) in a *nphp-4(tm925)* genetic background using an *unc-58* co-CRISPR strategy (84). Cas9 enzyme (IDT, #1081058), tracrRNA (IDT, #1072533), and custom synthesized crRNA were obtained from Integrated DNA Technologies. Suitable PAM sites were selected based on Azimuth 2.0 scores (85) and distance from the desired edit (<10 nucleotides). crRNA are listed in Supplementary Material, Table S3. Injection mixes were prepared on ice as follows: 1 μl crRNA (0.3 nmol/μl), 1 μl tracrRNA (0.425 nmol/μl), 0.25 μl *unc-58* crRNA (1 nmol/μl), 0.25 μl *unc-58* ssODN (500 ng/μl), 0.5 μl each variant specific ssODN (1 μg/μl), 2 μl 1 M KCl, 0.4 μl HEPES (200 mM, pH 7.4), 0.2 μl Cas9 (10 μg/μl) and RNAse-free water up to 10 μl. The injection mix was mixed gently, centrifuged at ~15 000 *g* for 2 min, and incubated at 37°C for 15 min before injection. All Unc F1 were screened in pools of three hermaphrodites and engineered alleles were detected with variant specific PCR primers. All primers are listed in Supplementary Material, Table S4. The CRISPR efficiency (defined as the percent of F1 pools that were positive for the edit) varied from 1% to 35% with an average 15%. One CRISPR mutant was isolated and characterized for each variant. Accuracy of the engineered variants was confirmed with Sanger sequencing. The co-CRISPR marker, *unc-58,* was also sequenced and unintended *unc-58* mutations (86) were outcrossed.

### *C. elegans* quantitative phenotyping assays

Assays to assess cilia structure and function were performed with young adult hermaphrodites (57). The phenotypic assays were performed blinded to genotype with at least three independent biological replicates. Quantitative dye filling assays were performed with DiO (Invitrogen, D275) and dye uptake of phasmid (tail) neurons was assessed on a wide-field epifluorescence microscope. For each variant, dye filling in 125–145 worms was quantified. Roaming activity of worms was quantified by placing a single young adult hermaphrodite on a fully seeded NGM plate for 20 h at 20°C. A 5 × 5 mm grid was used to count the number of squares the worm entered. The roaming activity of 30 worms was quantified for each variant. Values were normalized to wild-type (N2) for each replicate. Chemotaxis plates were prepared 16–24 h before the assay was performed (9 cm petri dishes with 10 ml of chemotaxis agar: 2% agar, 5 mM KPO_4_ pH6, 1 mM CaCl_2_, 1 mM MgSO_4_). Two points were marked at opposite sides of the plate 1.5 cm from the edge and 1 μl of 1M sodium azide (Sigma, S2002) was applied to the spots. Then 1 μl of ethanol (Honeywell, 32294) or 1:200 benzaldehyde (Sigma, B1334) diluted in ethanol was added to the spots. Young adult hermaphrodites were washed three times in M9 (22 mM KH_2_PO_4_, 42 mM N_a2_HPO_4_, 85.5 mM NaCl, 1 mM MgSO_4_) and once with deionized water and 50–300 worms were placed in the centre of the plate and excess water was removed. After 1 h the worms were counted. The chemotaxis index was calculated as follows: *(b−c)/n* where *b* is the number of worms within 1.5 cm of the benzaldehyde spot, *c* is the number of worms within 1.5 cm of the ethanol control, and *n* is the total number of worms on the plate. For each variant a total 15–25 assays were performed.

### Integration of phenotypic data to predict variant pathogenicity

The results from the dye filling, roaming and chemotaxis assays were consolidated to generate a value to predict variant pathogenicity. Averages from each assay were normalized to the *nphp-4(∆)* control (with a maximum value of 1.0 for each assay). These averages were then summed to generate the integrated phenotypic score. The *nphp-4(∆)* control received a score of 3.0 whereas the *mks-3* null allele received a score of 1.38. Variants that scored <2.5 were predicted to be pathogenic.

### Generating transgenic worms expressing extrachromosomal MKS-3::GFP

*mks-3::gfp* transgenes were generated with PCR-based fusion (87) of *mks-3* gDNA (including 485 bp of 5′ UTR sequence) with GFP and the *unc-54* 3′ UTR (pPD95_77, a gift from Andrew Fire, Addgene plasmid #1495). All primers are listed in Supplementary Material, Table S4. *mks-3(tm2547)* hermaphrodites were injected with 0.25 ng/μl *mks-3::gfp* and 100 ng/μl coel::dsRed (a gift from Piali Sengupta, Addgene plasmid #8938) to generate extrachromosomal arrays (1–7 lines each). PCR was used to confirm the presence of *mks-3::gfp* transgene in the stable extrachromosomal arrays.

### *C. elegans* wide-field imaging and quantification of fluorescence

Young adult hermaphrodites were immobilized on 4% agarose pads in 40 mM tetramisole (Sigma, L9756). Images were acquired with a 100× (1.40 NA) oil objective on an upright Leica DM5000B epifluorescence microscope and captured with an Andor iXon+ camera. Image analysis was performed with FIJI/ImageJ (NIH). MKS-3::GFP fluorescence was quantified as previously described (26)*.* Briefly, a 40 × 40 pixel box was drawn around a TZ pair and the integrated signal intensity was measured. The box size was increased by one pixel in each direction and the signal intensity of this 42 × 42 pixel box was used to calculate the background fluorescence. Background fluorescence was subtracted and values were normalized to wild-type.

### Transmission electron microscopy

Young adult hermaphrodites were processed as previously described (57). Briefly, worms were fixed in 2.5% glutaraldehyde (Merck) in Sørensen's phosphate buffer (0.1 M, pH 7.4) for 48 h at 4°C, post-fixed in 1% osmium tetroxide (EMS) for 1 h, and dehydrated through an increasing ethanol gradient. Samples were treated with propylene oxide (Sigma) and embedded in EPON resin (Agar Scientific) for 24 h at 60°C. Serial, ultra-thin (90 nm) sections of the worm nose tissue were cut using a Leica EM UC6 Ultramicrotome, collected on copper grids (EMS), stained with 2% uranyl acetate (Agar Scientific) for 20 min followed by 3% lead citrate (LabTech) for 5 min, and imaged on a Tecnai 12 (FEI software) with an acceleration voltage of 120 kV.

### Cell culture

Human hTERT-immortalized retinal pigmentary epithelial (hTERT-RPE1, American Type Culture Collection; ATCC) wild-type and crispant cell-lines were grown in Dulbecco’s minimum essential medium (DMEM)/Ham’s F12 medium supplemented with GlutaMAX (Gibco #10565018) and 10% foetal bovine serum (FBS). For selected experiments involving cilia, cells following passage were serum-starved in DMEM/F-12 media containing 0.2% FBS. Cells were cultured in an incubator at 37°C with 5% CO2.

### TMEM67 cloning, plasmid constructs and transfections

Full-length *H. sapiens* TMEM67 isoform 1 (RefSeq JF432845, plasmid ID HsCD00505975, DNASU Plasmid Repository) was cloned into pENTR223. The ORF was Gateway cloned into a C-terminal GFP-tagged Gateway pcDNA-DEST47 vector (ThermoFisher Scientific), sequence verified, and sub-cloned into pcDNA3.1 myc/HisA vector with HiFi cloning (New England Biolabs). The construct was also engineered to contain an endogenous Kozak sequence prior to the start site, the first 30 nucleotides of the main transcript that were missing from the DNASU sequence, and a GS linker between the ORF and myc tag. A QuikChange II XL Site-Directed Mutagenesis Kit (Agilent) was used according to the manufacturer’s protocol to generate TMEM67 variants. Primer sequences are listed in Supplementary Material, Table S5. The final constructs were verified by sequencing. Cells at 80% confluency were transfected with plasmids using Lipofectamine 2000 (Life Technologies Ltd.) as described previously (61).

### CRISPR/Cas9 genome editing in cell culture

GFP-expressing pSpCas9(BB)-2A-GFP (PX458) was a gift from Feng Zhang, Addgene plasmid #48138. Three crRNAs targeting human TMEM67 (RefSeq NM_153704.5) were designed using Benchling (https://benchling.com), selected for the highest ranking on- and off-target effects. crRNAs were ordered as HPLC-purified oligos from Integrated DNA Technologies in addition to Alt-R CRISPR-Cas9 tracrRNA-ATTO550 conjugates. crRNA sequences are listed in Supplementary Material, Table S6. Lyophilised pellets were resuspended in Tris-EDTA (TE) buffer (Qiagen) to give 100 mM stocks. crRNA and tracrRNA were mixed (1:1), and incubated in nuclease-free duplex buffer (30 mM HEPES, pH 7.5, 100 mM potassium acetate) to make 300 nM guide RNA master mixes. Before transfecting into cells the crRNA:tracrRNA duplexes were incubated with Lipofectamine 2000 at an RNA:Lipofetamine ratio of 2:1 in 200 μl/well Opti-MEM for 20 min. One millilitres of media from 6-well plate wells was removed, the transfection reagents applied, and the cells incubated overnight. Media was changed to fresh DMEM/F-12 with 10% FBS after 16 h, and cells incubated for 48 h. Following transfection, FACS was performed to enrich cells expressing GFP and to produce clonal populations. Ninety-six well plates (Corning) were treated with 200 μl of 4% bovine serum albumin (BSA) per well for 1 h. Wells were then filled with 100 μl of filter-sterilized collection buffer (20% FCS, 1% penicillin–streptomycin, 50% conditioned media, 29% fresh DMEM/F-12 media). Transfected cells were prepared for FACS by removing media, washing in PBS, and treating with trypsin for 5 min before resuspending in filter-sterilized sorting buffer (1× Ca^2+^/Mg^2+^-free PBS, 5 mM EDTA, 25 mM HEPES pH 7.0). A 70 μm filter was used to disperse cells into 4% BSA-treated polystyrene FACS tubes. A BD Influx 6-way cell sorter (BD Biosciences) was used to index sort GFP-positive cells, calibrated against un-transfected control cells. When an abundance of GFP-positive cells were present, the top 5% were targeted for index sorting. After sorting, cells were incubated for 3 weeks at 37°C with 5% CO2, with weekly checks for growing colonies.

### PCR and sequence validation of crispant cell-lines

To extract DNA from colonies within the 96-well plates, cells were washed with 1× Ca^2+^/Mg^2+^-free PBS and resuspended in 50 μl of DirectPCR Lysis Reagent (Viagen Biotech) containing 0.4 mg/ml Proteinase K (Sigma, # P4850). Suspensions were incubated at 55°C for 5 h, followed by 85°C for 45 min. One microlitre of DNA extracts were used in PCR reactions. Primers are listed in Supplementary Material, Table S7. Variants were identified by Sanger sequencing (GeneWiz Inc.) followed by analysis using the Synthego ICE v2 (88). The following clones were chosen for further study: clone 40, heterozygous for c.369delC (p.Glu124Lysfs^*^12); and clone 16 carrying biallelic variants [c.519delT]+[ c.519dupT] ([p.Cys173Trpfs^*^20]+[ p.Glu174^*^]). All variants were predicted to result in nonsense mediated decay by the Ensembl Variant Effect Predictor (89). Clone 21 was a negative control cell-line that was mock-transfected, underwent FACS, but was verified to carry wild-type *TMEM67*.

### Whole cell extract preparation and western immunoblotting

Whole cell extracts containing total soluble proteins were prepared from hTERT-RPE1 cells that were transiently transfected with 1.0 μg plasmid constructs in 90 mm tissue culture dishes, or scaled down as appropriate. Ten micrograms total soluble protein was analysed by SDS-PAGE (4–12% polyacrylamide gradient) and western blotting performed according to standard protocols. Primary antibodies used: mouse anti-β actin (1:10000, clone AC-15, Abcam Ltd., Cambridge, UK); rabbit polyclonal anti-TMEM67 (1:500, 13975-1-AP; ProteinTech Inc., Rosemont, IL, USA); goat anti-ROR2 (1:1000, AF2064; R&D Systems Inc., Minneapolis, MN, USA). Appropriate HRP-conjugated secondary antibodies (Dako UK Ltd.) were used (final dilutions of 1:10 000–25 000) for detection by the enhanced chemiluminescence ‘Femto West’ western blotting detection system (Thermo Fisher Scientific Inc., Rockford, IL, USA) and visualized using a ChemiDoc MP imaging system (BioRad Inc., Hercules, CA, USA). Ratios of active phosphorylated ROR2 : unphosphorylated ROR2 isoforms were calculated by quantitating band intensity using ImageLab 5.2.1 software (BioRad Inc.) for three biological replicates, as described previously (61).

### Statistical analyses

All *C. elegans* statistical analyses were performed in Microsoft Excel with the Real Statistics Resource Pack Version 7.2 (www.real-statistics.com). A Shapiro–Wilk test determined if data were normally distributed. Statistical significance of normally distributed datasets was determined with an ANOVA followed by Tukey’s *post hoc* (chemotaxis and GFP quantification) or Kruskal–Wallis followed by Dunn’s (roaming) for non-parametric datasets. Statistical significance of dye filling was determined using a Kruskal–Wallis followed by Schaich–Hammerle *post hoc* test using a chi-squared distribution. For cell culture results, a normal distribution of data were confirmed using the Kolmogorov–Smirnov test (GraphPad Prism). Pairwise comparisons were analysed with Student's two-tailed t-test using InStat (GraphPad Software Inc.). ^*^, ^*^^*^, and ^*^^*^^*^ refer to *P*-values of <0.05, <0.01 and <0.001, respectively.

## Supplementary Material

Supplemental_Lange_ddab344
