# Supplementary material for: Interpreting ciliopathy-associated missense variants of uncertain significance (VUS) in Caenorhabditis elegans
Source: Hum Mol Genet. 2021 Nov 20;31(10):1574–87. doi: 10.1093/hmg/ddab344 (PMC9122650; doi:10.1093/hmg/ddab344)
Supplement: Supplemental_Lange_ddab344 [file supplemental_lange_ddab344.pdf]

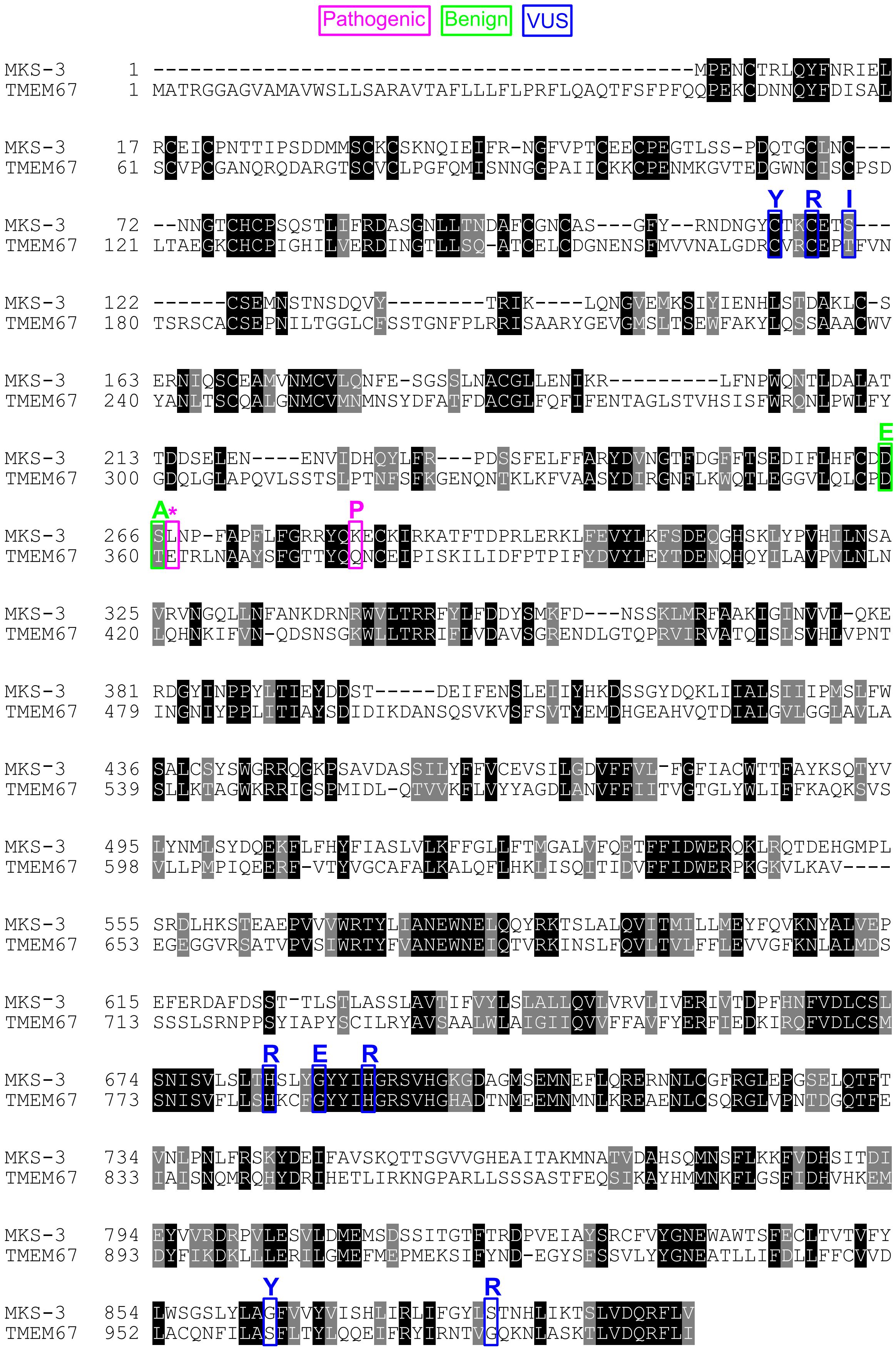

Figure S1. TMEM67 is conserved from humans to worms.

Protein alignment between human TMEM67 (NP_714915) and *C. elegans* MKS-3 (NP_495591.2). Conservation of identical (black) and similar (grey) amino acids are highlighted. Amino acids that were mutated in this study are labelled green (benign), magenta (pathogenic), and blue (VUS). Amino acid sequences were aligned using Clustal Omega and the figure was generated using BoxShade 3.21.

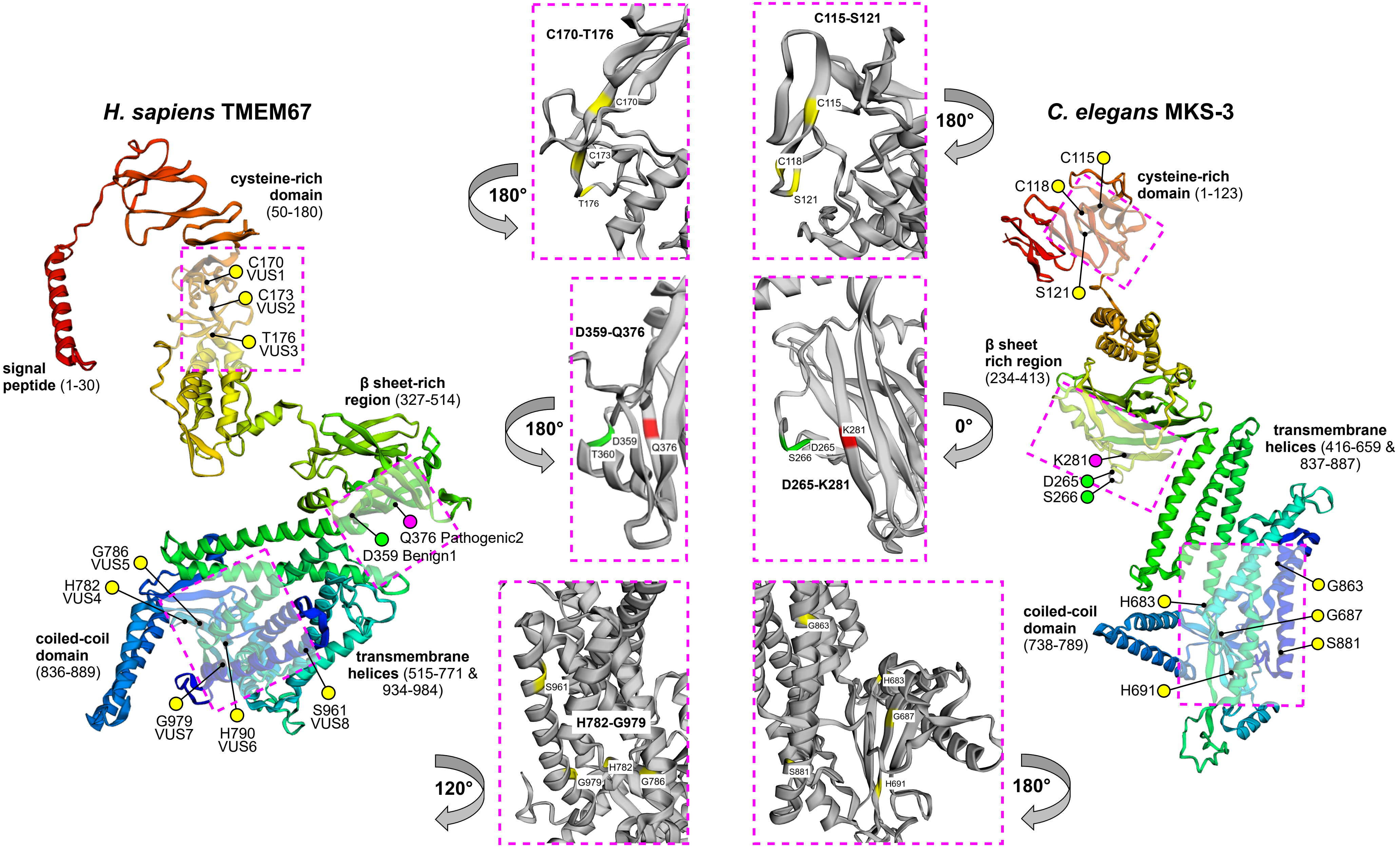

Figure S2. RaptorX predicted structures of TMEM67 and MKS-3

Ribbon diagrams of proteins are rainbow-coloured (red at N-terminus to dark blue at C-terminus) with variants indicated (red, known pathogenic; yellow, VUS; green, known benign). Insets highlight amino acids analyzed in this study.

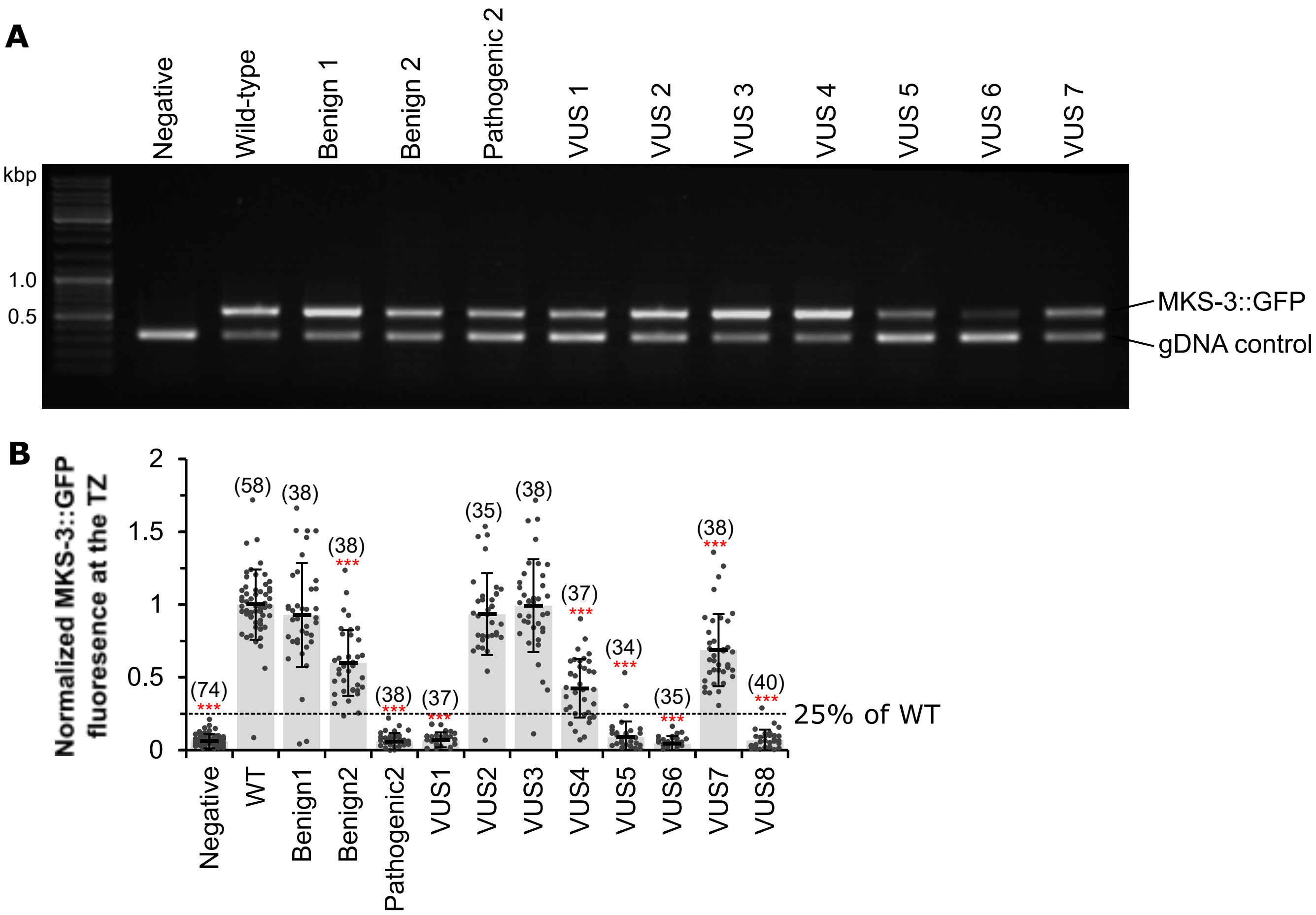

Figure S3. Transgenic MKS-3::GFP

A) PCR products after DNA gel electrophoresis. The upper band is specific to *mks-3::gfp*. The lower band is a gDNA control. Non-injected worms (Negative) do not contain the *mks-3::gfp* product while the transgenic strains do. PCR for *VUS8::gfp* is not shown.

B) Quantification of MKS-3::GFP levels at the transition zone. Background fluorescence was subtracted. Individual dots show each measurement while the bars show the average +/- the standard deviations. The total number of transition zone pairs measured is shown in brackets. If a measurements had more than 25% of the wild-type level (dashed line) we concluded that it was positive for MKS-3::GFP localization to the transition zone. Statistical significance according to a one way ANOVA followed by Tukey's *post hoc* test and is relative to the wild-type control. *** Indicates a p-value < 0.001.

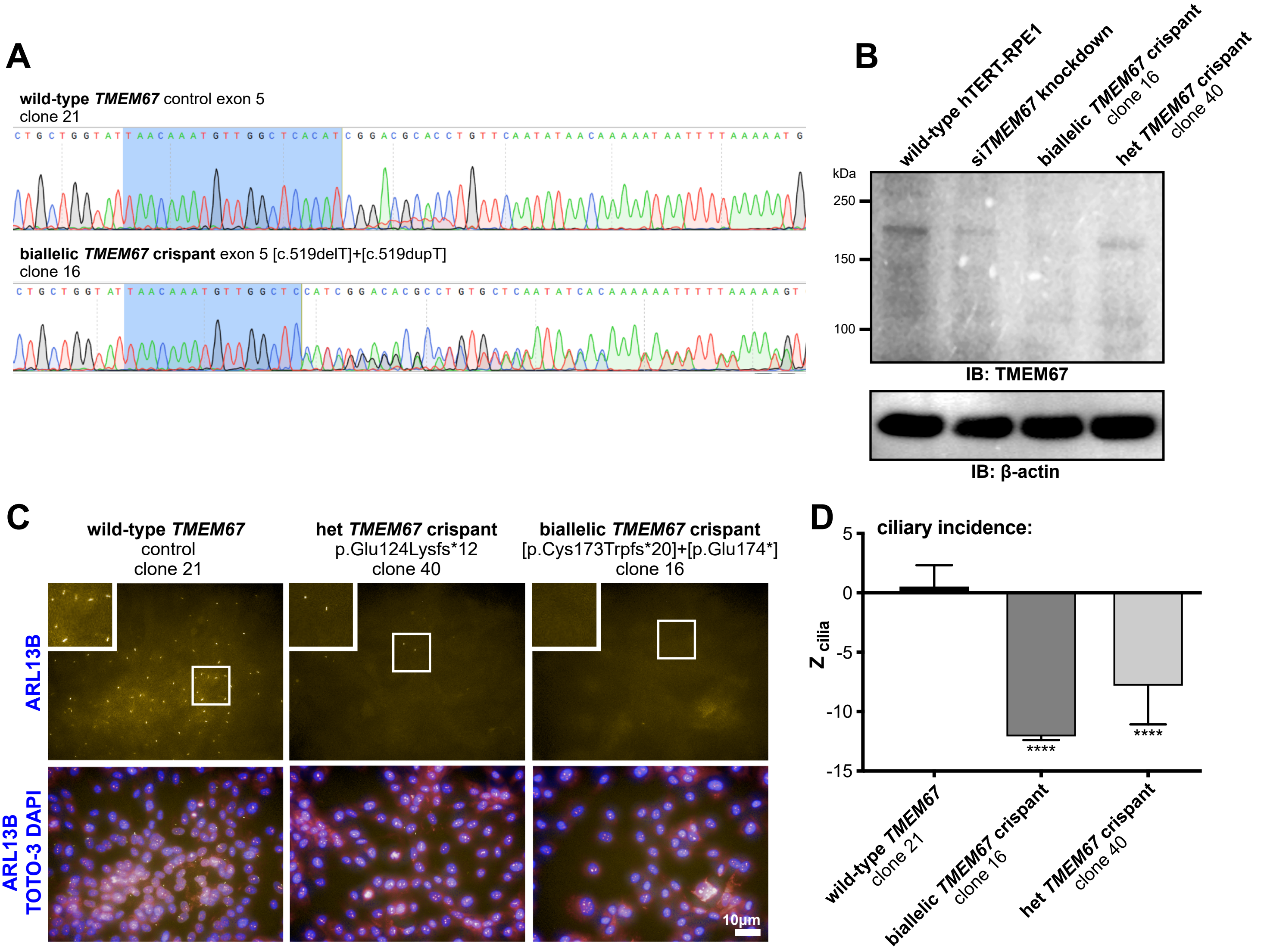

Figure S4. Characterization of *TMEM67* crispant

A) Sanger sequencing electropherograms comparing *TMEM67* exon 5 sequence between wild-type negative control cell-line clone 21 and the bi-allelic crispant cell-line clone 16. Highlighted sequence in blue indicates the guide RNA sequence used for targeting exon 5. Sequence analysis reveals a one base-pair deletion on one allele and a one base-pair insertion at the same position on the other allele corresponding to biallelic frameshift variants: c.519delT, p.(Cys173Trpfs*20) and c.519dupT, p.(Glu174*).

B) Western blotting of protein lysates from untreated wild-type hTERT-RPE-1, wild-type hTERT-RPE-1 following siRNA knockdown of *TMEM67*, the bi-allelic *TMEM67* crispant clone 16 and the heterozygous *TMEM67* crispant clone 40, with beta-actin as a loading control. A band visible at ca. 200kDa likely corresponds to post-translationally modified TMEM67 (expected molecular weight 112kDa). Levels of TMEM67 expression are most reduced in the bi-allelic knockout crispant clone 16 (no band visible), with decreased levels observed in the heterozygous crispant clone 40 and the siRNA TMEM67 knockdown compared to wild-type.

C) hTERT-RPE-1 cells imaged using an "Operetta" (Perkin-Elmer) high-content imaging system, with representative images from Harmony/Columbus software cilia recognition protocol “find spots”. Images show cells stained for the ciliary membrane protein ARL13B (gold), nuclei with DAPI (blue) and cytoplasm with TOTO3 (pink). Significantly fewer cilia were present in the heterozygous *TMEM67* crispant clone 40 compared to wild-type hTERT-RPE-1, and no cilia are visible in the bi-allelic *TMEM67* crispant clone 16. Frames indicate the position of magnified insets. Scale bar = 10mM.

D) Bar graphs showing mean robust z score for % ciliated cells (z cilia) for wild-type hTERT-RPE-1 (+0.60), the bi-allelic *TMEM67* crispant clone 16 (-12.15) and the heterozygous *TMEM67* crispant clone 40 (-7.89). Ciliary incidence is significantly decreased (z cilia < -2.0) in both crispant clones compared to wild-type.

Figure S5. Prediction of deleteriousness of missense alleles using *in silico* analysis

For each nonsynonymous variant, the text colour denotes if the prediction is tolerated/benign (green), deleterious/damaging (red), or possibly damaging (black). Using these prediction tools we ranked the variants for their overall predicted deleteriousness (1 = benign, 11 = most severe/pathogenic). Variants that are predicted to be more deleterious/damaging are assumed to correlate with disease pathogenesis. The different analyses consistently revealed Benign1 and Benign2 (green background) as benign and the least damaging of all 11 missense mutations. Pathogenic2 (gray background) is identified as deleterious by four of the five prediction tools and ranks as the 3rd most deleterious variant. Overall the *in silico* predictions suggest all eight VUS alleles are likely deleterious/damaging. The only exceptions are VUS3/8 (predicted as probably not damaging by PolyPhen-2 and CADD) and VUS7 (predicted to be tolerated by SIFT).

TMEM67[a]	Clinical Significance	MISTIC[b]	SIFT[c]	Poly-Phen2[d]	CADD[e]	REVEL[f]	Overall Rank
D359E	Benign1	0.293	0.27	0.25	17.0	0.338	1
T360A	Benign2	0.437	0.18	0.55	23.2	0.476	2
Q376P	Pathogenic2	0.965	0.14	0.998	26.0	0.935	9
C170Y	VUS1	0.926	0	1	27.1	0.889	6
C173R	VUS2	0.955	0	1	27.2	0.926	10
T176I	VUS3	0.782	0.03	0.681	24.2	0.596	4
H782R	VUS4	0.735	0	0.959	25.3	0.927	5
G786E	VUS5	0.746	0	1	32.0	0.919	8
H790R	VUS6	0.962	0	1	25.7	0.988	11
G979R	VUS7	0.901	0.25	1	28.8	0.958	7
S961Y	VUS8	0.741	0.02	0.495	24.8	0.781	3

a. Amino acid residues correspond to TMEM67 reference sequence NP_714915.
b. MISTIC(MISsense deleTeriousness predICtor) values are from 0-1 with >0.5 being deleterious (Chennen et al. 2020).
c.SIFT(Sorting Intolerant from Tolerant) scores probability of deleteriousness with <0.05 considered significant (Sim et al. 2012). Scores were calculated using an alignment of human TMEM67 with orthologs from mouse, rat, zebrafish, and nematodes.
d. PolyPhen-2(Polymorphism Phenotyping v2) scores range from 0-1 with >0.908 being probably damaging, > 0.446 ≤ 0.908 possibly damaging, and ≤ 0.446 benign (Adzhubei et al. 2010).
e. CADD(Combined Annotation Dependent Depletion) v1.6 phred scores range from 1-99 with higher scores being more deleterious (Rentzsch et al. 2019). We used a cut off of 25.0 because this was the score between the known benign and pathogenic scores.
f. REVEL(Rare Exome Variant Ensemble Learner) scores range from 0-1 with >0.5 being likely pathogenic (Ioannidis et al. 2016).

References

Adzhubei, Ivan A., Steffen Schmidt, Leonid Peshkin, Vasily E. Ramensky, Anna Gerasimova, Peer Bork, Alexey S. Kondrashov, and Shamil R. Sunyaev. 2010. “A Method and Server for Predicting Damaging Missense Mutations.” *Nature Methods* 7 (4): 248–49.

Chennen, Kirsley, Thomas Weber, Xavière Lornage, Arnaud Kress, Johann Böhm, Julie Thompson, Jocelyn Laporte, and Olivier Poch. 2020. “MISTIC: A Prediction Tool to Reveal Disease-Relevant Deleterious Missense Variants.” *PloS One* 15 (7): e0236962.

Ioannidis, Nilah M., Joseph H. Rothstein, Vikas Pejaver, Sumit Middha, Shannon K. McDonnell, Saurabh Baheti, Anthony Musolf, et al. 2016. “REVEL: An Ensemble Method for Predicting the Pathogenicity of Rare Missense Variants.” *American Journal of Human Genetics* 99 (4): 877–85.

Rentzsch, Philipp, Daniela Witten, Gregory M. Cooper, Jay Shendure, and Martin Kircher. 2019. “CADD: Predicting the Deleteriousness of Variants throughout the Human Genome.” *Nucleic Acids Research* 47 (D1): D886–94.

Sim, Ngak-Leng, Prateek Kumar, Jing Hu, Steven Henikoff, Georg Schneider, and Pauline C. Ng. 2012. “SIFT Web Server: Predicting Effects of Amino Acid Substitutions on Proteins.” *Nucleic Acids Research* 40 (Web Server issue): W452-7.

Table S1. Variants analyzed in this study

	Variant	Clinical Significance	GRCh37	cDNA	Protein	Additional information	Conditions	Publications
Benign1	D359E	---	Chr8:94794634	NM_153704.6:c.1077C>G	p.(Asp359Glu)			
Benign2	T360A	Likely Benign	Chr8:94794635	NM_153704.6:c.1078A>G	p.(Thr360Ala)		Joubert syndrome	Consugar, et al. (2015) Genetics in Medicine 17 (4): 253–61.
Pathogenic1	E361*	Pathogenic	Chr8:94794638	NM_153704.6:c.1081G>T	p.(Glu361Ter)	in trans with 5 nt insertion/frameshift	Joubert syndrome	Fleming, et al. (2017) CJASN 12 (12), 1962–1973.
Pathogenic2	Q376P	Pathogenic	Chr8:94794684	NM_153704.6:c.1127A>C	p.(Gln376Pro)	Homozygous	Meckel syndrome	Smith et al. (2006) Nature Genetics 38 (2): 191–96.
VUS1	C170Y	VUS*	Chr8:94777684	NM_153704.5:c.509G>A	p.(Cys170Tyr)	identified in trans with an established pathogenic splice variant (c.224-2A>T)	Meckel syndrome	This study.
VUS2	C173R	VUS	Chr8:94777644	NM_153704.6:c.517T>C	p.(Cys173Arg)	Compound heterozygote with S312P	Joubert syndrome	Huynh, et al. (2018) Clinical Case Reports 6(11), 2189–2192.
VUS3	T176I	VUS*	Chr8:94777654	NM_153704.6:c.527C>T	p.(Thr176Ile)	identified *in trans* with an established pathogenic variant	Meckel syndrome	This study.
VUS4	H782R	VUS	Chr8:94817012	NM_153704.4:c.2345A>G	p.(His782Arg)		COACH	Brancati, et al. (2009) Human Mutation 30 (2): E432–42.
VUS5	G786E	VUS	Chr8:94817024	NM_153704.6:c.2357G>A	p.(Gly786Glu)	Biallelic with nonsense allele (E848*)	Meckel syndrome	Iannicelliet al. (2010) Human Mutation 31(5), E1319–E1331.
VUS6	H790R	VUS*	Chr8:94817036	NM_153704.5:c.2369A>G	p.(His790Arg)	Identified heterozygous in a parent	Meckel syndrome	This study.
VUS7	G979R	VUS*	Chr8:94828627	NM_153704.5:c.2935G>A	p.(Gly979Arg)	identified in trans with an established pathogenic splice variant(c.507-3C>A)	Meckel syndrome	This study.
VUS8	S961Y	VUS/Likely pathogenic	Chr8:94827650	NM_153704.5:c.2882C>A	p.(Ser961Tyr)	identified *in trans* with an established pathogenic variant, no functional evidence	Meckel Syndrome	Szymanska, K., et al. (2012). Cilia, 1 (18).

*VUS1(C170Y), VUS3(T176I), VUS6(H790R), and VUS7(G979R) were identified from whole exome sequencing of suspected Meckel syndrome feotuses.

Table S2. Worm strains

	Strain	Genotype	Details
Controls	N2	Wild-type	
		nphp-4(tm925) V	
		mks-3(tm2547) II	949 bp deletion
		mks-3(tm2547) II; nphp-4(tm925) V	
CRISPR mutants	OEB934	*mks-3(oq123[K281P]) II; nphp-4(tm925) V*	Pathogenic 2, K281P
	OEB935	*mks-3(oq124[D265E]) II; nphp-4(tm925) V*	Benign 1, D265E
	OEB941	*mks-3(oq130[L267*]) II; nphp-4(tm925) V*	Pathogenic 1, L267*
	OEB942	*mks-3(oq131[S266A]) II; nphp-4(tm925) V*	Benign 2, S266A
	OEB943	*mks-3(oq132[C115Y]) II; nphp-4(tm925) V*	VUS1, C115Y
	OEB944	*mks-3(oq133[C118R]) II; nphp-4(tm925) V*	VUS2, C118R
	OEB955	*mks-3(oq134[H683R]) II; nphp-4(tm925) V*	VUS4, H683R
	OEB956	*mks-3(oq135[G687E]) II; nphp-4(tm925) V*	VUS5, G687E
	OEB957	*mks-3(oq136[H691R]) II; nphp-4(tm925) V; unc-58(oq146) X*	VUS6, H691R
	OEB1018	*mks-3(oq136[H691R]) II; nphp-4(tm925) V*	VUS6, H691R, outcrossed 1x
	OEB980	*mks-3(oq139[S121I]) II; nphp-4(tm925) V*	VUS3, S121I
	OEB981	*mks-3(oq140[S881R]) II; nphp-4(tm925) V; unc-58(oq147) X*	VUS7, S881R
	OEB1019	*mks-3(oq140[S881R]) II; nphp-4(tm925) V*	VUS7, S881R, outcrossed 1x
	OEB1001	*mks-3(oq145[G863Y]) II; nphp-4(tm925) V*	VUS8, G863Y
Extrachromosomal Arrays	OEB990	*mks-3(tm2547) II; oqEx122[mks-3p::mks-3::gfp + coel::dsRed]*	Wild-type
	OEB991	*mks-3(tm2547) II; oqEx123[mks-3p::mks-3(oq124)::gfp + coel::dsRed]*	Benign 1, D265E
	OEB992	*mks-3(tm2547) II; oqEx124[mks-3p::mks-3(oq131)::gfp + coel::dsRed]*	Benign 2, S266A
	OEB993	*mks-3(tm2547) II; oqEx125[mks-3p::mks-3(oq123)::gfp + coel::dsRed]*	Pathogenic 2, K281P
	OEB994	*mks-3(tm2547) II; oqEx126[mks-3p::mks-3(oq132)::gfp + coel::dsRed]*	VUS1, C115Y
	OEB995	*mks-3(tm2547) II; oqEx127[mks-3p::mks-3(oq133)::gfp + coel::dsRed]*	VUS2, C118R
	OEB996	*mks-3(tm2547) II; oqEx128[mks-3p::mks-3(oq139)::gfp + coel::dsRed]*	VUS3, S121I
	OEB997	*mks-3(tm2547) II; oqEx129[mks-3p::mks-3(oq134)::gfp + coel::dsRed]*	VUS4, H683R
	OEB998	*mks-3(tm2547) II; oqEx130[mks-3p::mks-3(oq135)::gfp + coel::dsRed]*	VUS5, G687E
	OEB999	*mks-3(tm2547) II; oqEx131[mks-3p::mks-3(oq136)::gfp + coel::dsRed]*	VUS6, H691R
	OEB1000	*mks-3(tm2547) II; oqEx132[mks-3p::mks-3(oq140)::gfp + coel::dsRed]*	VUS7, S881R
	OEB1020	*mks-3(tm2547) II; oqEx133[mks-3p::mks-3(oq145)::gfp + coel::dsRed]*	VUS8, G863Y

Table S3. Worm crRNA sequences and repair templates

crRNA sequence	Allele	Mutation	ssODN Repair Template Sequence*	Strand
ATCCACGCACATGGTCACTA	---	*unc-58* co-CRISPR	attttgtggtataaaatagccgagttaggaaacaaatttttctttcagGT**TTT**TCTGTCGTTACCATGTGCGTGGATCTTGCGTCCACACATCTCAAGGCGTACTT	sense
AAAAGAATGCAAAATAAGAA	*oq123*	Pathogenic 2, K281P	TGAACCCATTTGCTCCATTTTTATTCGGAAGACGTTATCAA**CCA**GAGTGCAAGATCCGTAAGGCAACATTCACAGATCCACGTCTTGAGAGAAAATTATT	sense
CGAATAAAAATGGAGCAAAT	*oq124*	Benign 1, D265E	TTTTTCACTTCGGAAGACATATTTCTTCATTTTTGTGAT**GAG**TCCCTTAATCCATTCGCCCCATTTTTATTCGGAAGACGTTATCAAAAAGAATGCAAAAT	sense
	oq130	Pathogenic 1, L267*	TTTCACTTCGGAAGACATATTTCTTCATTTTTGTGATGACTCT**TGA**AATCCATTCGCCCCATTTTTATTCGGAAGACGTTATCAAAAAGAATGCAAAATAA	sense
	oq131	Benign 2, S266A	TTTTCACTTCGGAAGACATATTTCTTCATTTTTGTGATGAC**GCC**CTCAATCCATTCGCCCCATTTTTATTCGGAAGACGTTATCAAAAAGAATGCAAAAT	sense
GGCTTTTACAGAAATGACAA	*oq132*	VUS1, C115Y	gaatgaacaaacCATTTCGGAACACGACGTTTCACATTTCGT**ATA**GTATCCGTTATCGTTTCTGTAAAAGCCAGAAGCACAGTTTCCACAGAATGCATCA	antisense
	oq133	VUS2, C118R	atgtgaatgaacaaacCATTTCGGAACACGACGTTTC**ACG**CTTCGTGCACTATCCGTTATCGTTTCTGTAAAAGCCAGAAGCACAGTTTCCACAGAATGCA	antisense
TCTCTGACCCATTCTCTGTA	*oq134*	VUS4, H683R	CATCACCCTTTCCATGAACAGAACGACCATGAATATAGTATCCATAGAGGGA**GCG**GGTCAGAGAAAGGACActgaaattgatttaagccgtgaagaattt	antisense
	oq135	VUS5, G687E	CCAGCATCACCCTTTCCATGAACAGAACGACCATGAATATAGTA**CTC**ATAGAGGGAGTGGGTCAGAGAAAGGACActgaaattgatttaagccgtgaagaa	antisense
	oq136	VUS6, H691R	TCATTCCAGCATCACCCTTTCCATGAACAGAACGACC**ACG**GATGTAGTAACCATAGAGGGAGTGGGTCAGAGAAAGGACActgaaattgatttaagccgt	antisense
TGTGAATGAACAAACCATTT	*oq139*	VUS3, S121I	GCTTTTACAGAAATGACAACGGATATTGCACGAAATGTGAAACG**ATC**TGCTCTGAGATGgtttgttcattcacattttagtgttttctttttggaatact	sense
ACAAGTAACCGAAAATCAAA	*oq140*	VUS7, S881R	TGCTGGTTTTGTTGTGTATGTTATTTCTCACTTGATCCGCCTCATCTTCGGATACCTC**CGT**ACGAATCATTTGATTAAGACGAGTTTAGTAGATCAACGA	sense
TCTGGATCTTTATATCTTGC	*oq145*	VUS8, G863Y	ATGTTTAACAGTTACAGTCTTCTATTTATGGTCTGGATCTTTATACCTCGCT**TAC**TTTGTTGTGTATGTTATTTCTCACTTGATCCGTTTGATTTTCGGT	sense

*Red text indicates mutations and the codon with the amino acid substitution is in bold and underlined. Introns are in lower case.

Table S4. Worm sequencing/PCR primers

ID	Name	Sequence	Purpose
NL132	F35D2.4_R+2720	tggttcaagtcctcggaatc	Genotype *tm2547*
NL446	F35D2.4_F+374	tgcccttcacaaagcactct	Genotype *tm2547*
NL447	F35D2.4_F+1537	catcatattcaattgttaaatacggtg	Genotype *tm2547*
KL154	mks-3.F-2	ctATGCCTGAAAATTGTACGAG	Genotype *oq132, oq133*
KL155	mks-3.R+718	tcaaaatgaaacCTCGCAAC	Genotype *oq132, oq133, oq139*
KL156	mks-3.C115Y	CGACGTTTCACATTTCGTatAg	Genotype *oq132*
KL159	mks-3.C118R	AACACGACGTTTCACgcTTg	Genotype *oq133*
KL161	mks-3.F+798	TTCACTGAATGCGTGTGGAC	Genotype *oq123, oq124, oq130, oq131*
KL162	mks-3.R+1664	AGAGCTGACCAGAACAATGAC	Genotype *oq123, oq131*
KL163	mks-3.D265E	gGCgAATGGaTTaAGgGAc	Genotype *oq124*
KL164	mks-3.S266A	CATTTTTGTGATGACgCcCTc	Genotype *oq131*
KL165	mks-3.L267Ter	TAAAAATGGgGCgAATGGaTTtc	Genotype *oq130*
KL166	mks-3.K281P	CTTaCggATcTTGCAcTCTgg	Genotype *oq123*
KL176	mks-3.R.1123	AAATGGAGCAAATGGGTTC	Genotype *oq124, oq130, oq131*
KL177	mks-3.For-39	CAAATGCTCAGTTTCGTTCAC	Genotype *oq139*
KL178	mks-3.F+2936	TCGGGATCGACCAGTCTTG	Genotype *oq140*
KL179	mks-3.R+3649	caggagatcagtgcgaacg	Genotype *oq140*
KL181	mks-3.F+2125	TGCGAATGAATGGAATGAAC	Genotype *oq134, oq135*
KL182	mks-3.R+2814	CAGATGTAGTTTGTTTGGAGAC	Genotype *oq134, oq135, oq136*
KL184	mks-3.S881R-Rev	TAATCAAATGATTCGTacggAg	Genotype *oq140*
KL185	mks-3.H683R-Rev	ATAGTATCCATAgAGgGAgc	Genotype *oq134*
KL186	mks-3.G687E-For	GACCCAcTCcCTcTATGag	Genotype *oq135*
KL188	mks-3.H691R-For	cTATGGtTACTAcATcCg	Genotype *oq136*
KL199	mks-3.For+2532	TTCTCTGACCCATTCTCTG	Genotype *oq136*
KL200	S121I.Rev	caaacCATcTCaGAgCAgat	Genotype *oq139*
KL183	mks-3.G863Y-Rev	TACACAACAAAgtaAGCgAGg	Genotype *oq145*
KL230	unc-58. For+3669	GACTCGGAGATATCGTTGTGACTG	*unc-58* PCR
KL231	unc-58. Rev+4393	CGCGGAGTTCGTTATCCAGGAAG	*unc-58* PCR
KL232	unc-58. Rev+4367	CGCACATCATTCCATGTAAC	Sequencing primer
NL72	GFP For	AAGCTTGCATGCCTGCAGGTCGACTC	Amplify GFP
NL118	GFP Rev(D-1)	tcaccgtcatcaccgaaacg	Amplify GFP
KL229	mks-3.For-495	tgtctttgactaggcataacccaac	*mks-3::gfp* stitch (PCR1)
NL441	F35D2.4_F-1196	tggtaatttgctcagtgtttcaattg	*mks-3::gfp* stitch (PCR1)
NL443	mks-3_GFP_R1	GAGTCGACCTGCAGGCATGCAAGCTTaacaagaaatcgttgatctactaaactcgt	*mks-3::gfp* stitch (PCR1)
ST54	mks-3_-485_F	taggcataacccaacaatcaac	*mks-3::gfp* stitch (PCR2)
NL74	GFP Rev (D*)	GGAAACAGTTATGTTTGGTATATTGGG	*mks-3::gfp* stitch (PCR2)

Table S5. Site Directed Mutagenesis Primers

Target TMEM67 cDNA change	Target TMEM67 protein change	Primer Direction	Sequence (5' to 3')
c.509G>A	Cys170Tyr	Forward	gctttaggagacaggtacgtccgatgtgagc
		Complement	gctcacatcggacgtacctgtctcctaaagc
c.515G>A	Arg172Gln	Forward	aaatgttggctcacattggacgcacctgtctcc
		Complement	ggagacaggtgcgtccaatgtgagccaacattt
c.517T>C	Cys173Arg	Forward	caaatgttggctcacgtcggacgcacctgtc
		Complement	gacaggtgcgtccgacgtgagccaacatttg
c.527C>T	Thr176Ile	Forward	ctgctggtattaacaaatattggctcacatcggacgc
		Complement	gcgtccgatgtgagccaatatttgttaataccagcag
c.1077C>G	Asp359Glu	Forward	ccttgtctctgtctctggacaaagctgtaaaacac
		Complement	gtgttttacagctttgtccagagacagagacaagg
c.1078A>G	Thr360Ala	Forward	catttagccttgtctctgcgtctggacaaagctgtaa-
		Complement	ttacagctttgtccagacgcagagacaaggctaaatg
c.2935G>A	Gly979Arg	Forward	atgccaaattcttttgtcttactgtattacggatatatctaaaaatctcttgt
		Complement	acaagagatttttagatatatccgtaatacagtaagacaaaagaatttggcat
c.2882C>A	Ser961Tyr	Forward	tcttgttgtagatatgtaaggaagtatgctaaaataaaattttggcaagc
		Complement	gcttgccaaaattttattttagcatacttccttacatatctacaacaaga
c.1127A>C	Gln376Pro	Forward	gagataggaatctcacaatttggttggtaggttgttccaaatg
		Complement	catttggaacaacctaccaaccaaattgtgagattcctatctc

Primers were designed using the web-based QuikChange Primer Design Program (https://www.agilent.com/store/primerDesignProgram.jsp).

Table S6. TMEM67 Alt-R crRNAs

TMEM67 target exon	crRNA sequence	PAM	Specificity Score	Efficiency Score
2	CAGATGATCTCTAATAATGG	AGG	63.16	71.18
3	CCTAGTGACTTAACTGCCGA	AGG	90.6	63.58
5	TAACAAATGTTGGCTCACAT	CGG	64.11	71.28

Table S7. TMEM67 sequencing/PCR primers

Target	Primer name	Primer sequence (5' to 3')
Internal TMEM67	cDNA1-RV	AACAGTGCTCAGTCCAGCAG
Internal TMEM67	EXON8R	CACACATATTTCCAAGAGCTTGAC
Internal TMEM67	EX4-7R	TTGCAAACCATTCTGAAGTTAAAG
Internal TMEM67	EX4-7F	TTGTGAGCTCTGTGATGGAAA
Internal TMEM67	EXON3F	TGTCCCATTGGCCATATTTT
Internal TMEM67	EX27-3'UTR-R	ACTACACACAATGGGAAAACAGTA
Internal TMEM67	ISH2R	AAAAATATGGCAAACTAACCTGA
Internal TMEM67	CDNA5-RV	AAAAATATGGCAAACTAACCTGA
Internal TMEM67	EXONIC 2F	TGTAAAAAGTGCCCAGAAAACA
Internal TMEM67	EX27-3'UTR-F	TGTGTTGTGGATTTGGCTTG
Internal TMEM67	ISH3F	TCTTGGCTCCTTCATTGACC
Internal TMEM67	CDNA4-RV	TGGGTACCAAACCTCTCTGG
Internal TMEM67	ISH2F	TCGACAGTTCGTTGATTTATGC
Internal TMEM67	EXON20/21R	CCCACAACCTCCAAAAAGAA
Internal TMEM67	EX19R	GAGCTTATGCAAAAATTGTAGTGC
Internal TMEM67	ISH1R	AGACTGGCTGTTGGCATCTT
Internal TMEM67	CDNA2-RV	TCGAATTACTCTTGGCTGAGTTC
Internal TMEM67	ISH6F	TTTTGGCTGTGCCTGTGTTA
Internal TMEM67	ISH5R	GGAAGCAGCAACAAACTTCA
Internal TMEM67	TMEM67_1203_F	GGCTGTGCCTGTGTTAAACC
Internal TMEM67	TMEM67_1481_R	GACTGGCTGTTGGCATCTTTG
Internal TMEM67	TMEM67_1942_F	GTACGAAGTGCCACTGTTCCTG
Internal TMEM67	TMEM67_2459_R	GTCTGACCATCTGTGTTGGGT
Internal PX458	T7	TAATACGACTCACTATAGGG
Internal PX458	U6	GACTATCATATGCTTACCGT
Internal PX458	BGHR	TAGAAGGCACAGTCGAGG
Internal PX458	CMV-Forward	CGCAAATGGGCGGTAGGCGTG
Internal PX458	EGFP-C-For	CATGGTCCTGCTGGAGTTCGTG
Internal PX458	EGFP-C-REV	GTTCAGGGGGAGGTGTG
Internal PX458	EGFP-N	CGTCGCCGTCCAGCTCGACCA
Internal PX458	EXFP-R	GTCTTGTAGTTGCCGTCGTC
Internal PX458	F1ori-F	GTGGACTCTTGTTCCAAACTGG
Internal PX458	M13 F	TGTAAAACGACGGCCAGT
Internal PX458	pBR322ori-F	GGGAAACGCCTGGTATCTTT
Internal PX458	SP6	ATTTAGGTGACACTATAG
Internal PX458	T7	TAATACGACTCACTATAGGG
Internal PX458	SpCas9_1F	GCCAAGGTGGACGACAGCTT
Internal PX458	SpCas9_2F	ACCTACAACCAGCTGTTCGAGG
Internal PX458	SpCas9_3F	CAAGAACCTGTCCGACGCC
Internal PX458	SpCas9_4F	GTGAAGCTGAACAGAGAGGACCT

Internal PX458	SpCas9_5F	TTCATCGAGCGGATGACCAACT
Internal PX458	SpCas9_6F	CTGGGCACATACCACGATCTG
Internal PX458	SpCas9_7F	AACTTCATGCAGCTGATCCACGA
Internal PX458	SpCas9_8F	GGCAGCCAGATCCTGAAAGAAC
Internal PX458	SpCas9_9F	CGCCAAGCTGATTACCCAGAG
Internal PX458	SpCas9_10F	GTGGGAACCGCCCTGATCAA
Internal PX458	SpCas9_11F	TCGTGAAAAAGACCGAGGTGC
Internal PX458	SpCas9_12F	AGGACCTGATCATCAAGCTGC
Internal PX458	SpCas9_13F	GTCCGCCTACAACAAGCACC
Internal PX458	Cas9_14_F	GCTTCACTCTCCCCATCTCC
Internal PX458	Cas9_15_F	GCGCTCCGAAAGTTTCCTTT
TMEM67 CRISPR targets	Exon2_F	CCCTAGTCCCCGTAAATGGA
TMEM67 CRISPR targets	Exon3_F	CTGGTTCAAGCCATTCTGGT
TMEM67 CRISPR targets	Exon5_F	CCCTCCCTCTGTCTCATTCA
TMEM67 CRISPR targets	Exon 2_R	ACACATCCCAGACTGCTCTA
TMEM67 CRISPR targets	Exon3_R	GGCTGAGCAACATAACGAGA
TMEM67 CRISPR targets	Exon5_R	AACAGGCTACGGTACAGACT
